# Forecasting the evolution of the 2021 Tajogaite eruption, La Palma, with TROPOMI/PlumeTraj-derived SO_2_ emission rates

**DOI:** 10.1007/s00445-025-01803-6

**Published:** 2025-02-26

**Authors:** B. Esse, M. Burton, C. Hayer, G. La Spina, A. Pardo Cofrades, M. Asensio-Ramos, J. Barrancos, N. Pérez

**Affiliations:** 1 Centre for the Observation and Modelling of Earthquakes, Volcanoes and Tectonics, Department of Earth and Environmental Sciences, The University of Manchester, Manchester, UK; 2 Istituto Nazionale Di Geofisica E Vulcanologia, Catania, Italy; 3 HAMTEC for EUMETSAT, Darmstadt, Germany; 4 Instituto Volcanológico de Canarias (INVOLCAN), 38320 San Cristóbal de La Laguna, Tenerife, Canary Islands Spain; 5 Instituto Tecnológico y de Energías Renovables (ITER), 38600 Granadilla de Abona, Tenerife, Canary Islands Spain; 6 Grupo de Observación de La Tierra y La Atmósfera (GOTA), Universidad de La Laguna. Avda. Astrofísico Francisco Sánchez S/N, La Laguna, 38200 Tenerife, Spain

**Keywords:** Sulphur dioxide, Eruption forecasting, Tajogaite, TROPOMI

## Abstract

As global populations grow, the exposure of communities and infrastructure to volcanic hazards increases every year. Once a volcanic eruption begins, it becomes critical for risk managers to understand the likely evolution and duration of the activity to assess its impact on populations and infrastructure. Here, we report an exponential decay in satellite-derived SO_2_ emission rates during the 2021 eruption of Tajogaite, La Palma, Canary Islands, and show that this pattern allows a reliable and consistent forecast of the evolution of the SO_2_ emissions after the first third of the total eruption duration. The eruption ended when fluxes dropped to less than 6% of their fitted maximum value, providing a useful benchmark to compare with other eruptions. Using a 1-D numerical magma ascent model, we suggest that the exponentially decreasing SO_2_ emission trend was primarily produced by reducing magma chamber pressure as the eruption emptied the feeding reservoir. This work highlights the key role that satellite-derived SO_2_ emission data can play in forecasting the evolution of volcanic eruptions and how the use of magma ascent models can inform the driving mechanisms controlling the evolution of the eruption.

**Supplementary Information:**

The online version contains supplementary material available at 10.1007/s00445-025-01803-6.

## Introduction

The risks posed by volcanic eruptions globally is growing with time as populations expand and exposure increases, with even relatively small magnitude eruptions capable of significant disruption to proximal populations (Mani et al. 2021). Forecasting the onset and evolution of volcanic eruptions is key for effective volcano monitoring and minimisation of the risks posed by active volcanoes (Sparks 2003; Sparks et al. 2012; Kilburn 2018; Poland and Anderson 2020; Poland et al. 2020; Kilburn and Bell 2022). Previous work focused on forecasting the start of eruptions using (typically) seismic (Kilburn and Bell 2022), geodetic (Segall 2013; Biggs et al. 2022), thermal (Wright et al. 2004) and gas (Young et al. 1998; Duffell et al. 2003; Aiuppa et al. 2010) signals. However, once an eruption begins, we have access to much more information which could be used to provide forecasts on the evolution and likely cessation of the eruption. Previous examples of this include using lava effusion rates (Wadge 1981; Bonny and Wright 2017) and geodetic measurements (Charco et al. 2024); however, to date, this has not been applied to volcanic gas emissions.

Volcanic eruptions are driven by the exsolution and expansion of volatile species as magma ascends towards the surface (Sparks 1978), and the degree of coupling between exsolved gases and magma controls the style of eruptive activity, from explosive to effusive (Eichelberger et al. 1986; Klug and Cashman 1996; Burton et al. 2007b). Each volatile species has a unique magma solubility behaviour, and the chemical composition and emission rate of the gases produced during an eruption reflects the dynamics of magma storage, evolution and ascent (though often overprinted by additional processes such as interactions with a hydrothermal system). Measurements of the emission rate and composition of magmatic gases therefore provide unique insights into magmatic systems (Allard et al. 2005; Burton et al. 2007a, 2023; Aiuppa et al. 2007; McCormick Kilbride et al. 2016; Yip et al. 2022).

The primary target species for quantifying volcanic gas emissions is sulphur dioxide (SO_2_) due to its high concentration in volcanic emissions (after water and carbon dioxide) and low atmospheric concentration (Symonds et al. 1994). SO_2_ can also be measured remotely from both ground and space thanks to absorption features at ultraviolet and infrared wavelengths (Oppenheimer et al. 2011). Satellites are particularly useful for eruption monitoring as they do not require a dedicated ground-based network of instrumentation and are well suited to imaging large (100 s of km scale) volcanic clouds as they disperse in the atmosphere. The quality of satellite measurements has improved in recent years thanks to increased spatial resolution, for example from 13 × 24 km for the Ozone Monitoring Instrument (OMI, de Graaf et al. 2016) to 3.5 × 5.5 km for the TROPOspheric Monitoring Instrument (TROPOMI, Veefkind et al. 2012), and analysis algorithms (Theys et al. 2021).

In this paper, we use satellite-derived volcanic SO_2_ emission rates to forecast the evolution of the 2021 eruption of Tajogaite Volcano, part of the Cumbre Vieja volcanic system on the island of La Palma (Canary Islands, Spain). We fit an exponential decay model to the cumulative SO_2_ emissions to determine if it would have been possible to forecast the future evolution of activity at different stages of the eruption, finding consistent projections from 20 October onwards. We then use a model of magma ascent to explore the likely physical processes controlling the observed exponential decline in gas emissions. The availability of satellite SO_2_ imaging data globally allows this approach to be applied generally to past and future eruptions. Those showing similar temporal evolution would suggest a single draining magma reservoir supplying the eruption (Wadge 1981), while deviations from this model would suggest a more complex system supplying the activity. Although the analysis we present here was performed after the eruption ended, we calculated SO_2_ emission rates in near real-time and performed a simple linear decay forecast throughout the crisis, which were communicated to the relevant authorities (ITER and INVOLCAN) daily. This provides the framework to repeat this for future volcanic crises.

### Eruption summary

**Fig. 1:**
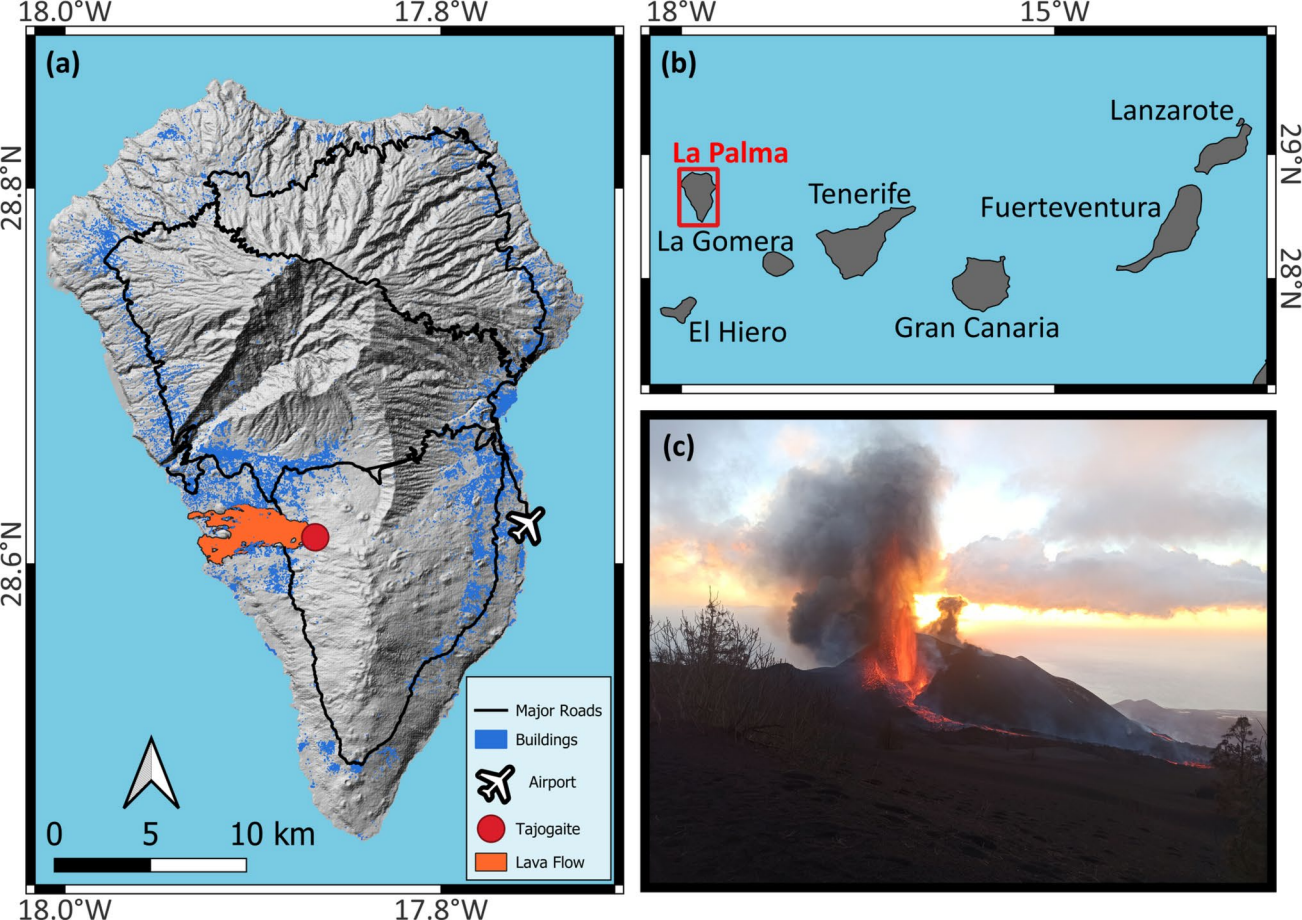
Overview of the Tajogaite eruption location. **a** A map of La Palma with the location of the eruption vent (red circle), lava flow footprint (orange), main roads (black lines) buildings (blue) and airport; **b** its location in the Canary Islands; and **c** an image of Tajogaite with a view of the summit vents, taken from the east on 2 December 2021 (image credit: Ana Pardo Cofrades). Elevation data is from the Shuttle Radar Topography Mission (SRTM) Elevation Dataset (NASA 2002). Lava flow footprint from Copernicus Emergency Management Service (2021). Building and road data taken from OpenStreetMap

The Cumbre Vieja rift system forms the main spine of the southern portion of La Palma (Fig. 1) and is the most volcanically active system in the Canaries, accounting for half of all eruptions in the last 500 years on the archipelago. These eruptions typically occur where magma follows pre-existing fractures in the edifice to the surface where they form multiple eruptive vents aligned in a fissure. The higher altitude vents are typically explosive while lower altitude vents tend to be effusive and produce lava flows. The durations of these eruptions range from 25 to 84 days, with the shortest being the most recent Teneguía eruption in 1971, which took place towards the southern end of Cumbre Vieja (Troll and Carracedo 2016).

The 2021 eruption of Tajogaite was well-monitored from the ground and space (Bonadonna et al. 2022; Carracedo et al. 2022; De Luca et al. 2022; Román et al. 2022; Romero et al. 2022; Burton et al. 2023), as well as through rapid petrological analyses (Pankhurst et al. 2022). The eruption was preceded by deep (25–35 km) seismic swarms detected since 2017, though with no measured deformation at that time (Torres-González et al. 2020). The seismic activity intensified in the week before the eruption began, with low-magnitude (typically < 2) seismicity in the week before the eruption tracing the path of magma from a depth of 10 km to the surface (D’Auria et al. 2022). Differential Interferometric Synthetic Aperture Radar (D-InSAR) and ground-based Global Navigation Satellite System (GNSS) stations showed pre-eruptive ground deformation from the emplacement of magma approximately 4.5 km below sea level from mid-August (De Luca et al. 2022). Geochemical monitoring of hot springs on the island also detected anomalies in the ^3^He/^4^He ratio of > 10 R_A_ (compared to an average of 9.8 R_A_ between 1991 and 2021) in 2020, associated with the intrusion of less-degassed magma at depth (Padrón et al. 2022).

The eruption began at 14:00 (local time) on 19 September 2021 with the opening of a rift on the western flank of the Cumbre Vieja ridge (28.61°N, 17.87°W, Fig. 1a), with initial activity characterised by lava fountaining and rapid Strombolian explosions, transitioning to more sustained fountaining over the following few days (Romero et al. 2022). A’a’ lava flows produced from the vent progressed to the west, eventually reaching the sea (~ 6 km away) and forming a lava delta, with a total area of roughly 12 km^2^ (Civico et al. 2022). The eruption also produced significant ashfall (2 × 10^7^ m^3^), blanketing the region surrounding the vent, particularly along the SW-NE axis (Bonadonna et al. 2022). The final volcanic edifice was 187 m high, reaching a total altitude of 1071 m asl (Civico et al. 2022). The eruption led to the evacuation of almost 8000 people, with lava burying close to 3000 buildings and destroying the main road (LP-2) connecting the northern and southern areas of the west of the island (Carracedo et al. 2022). Volcanic gas and aerosol emissions also impacted the local air quality, with measured SO_2_ and particulate concentrations on the island repeatedly breaking air quality thresholds from the European Commission and the World Health Organisation (Milford et al. 2023). The eruption lasted until 13 December (85 days), finishing with a strong explosion that produced an ash plume with an altitude of approximately 8 km asl (Romero et al. 2022).

## Data and methods

### TROPOMI

In this study, we utilised daily SO_2_ imagery from TROPOMI (Theys et al. 2017) to investigate the degassing behaviour throughout the Tajogaite eruption. TROPOMI is onboard the European Space Agency’s Sentinel-5P satellite in a sun-synchronous polar-orbit, providing daily (almost) global coverage (Veefkind et al. 2012). The SO_2_ imagery has a spatial resolution of 5.5 × 3.5 km^2^ at nadir (along-track × across-track, upgraded from 7.0 × 3.5 km^2^ from 6 August 2019).

TROPOMI provides daily snapshots of SO^2^ distribution in the atmosphere but does not reveal the gas source and plume altitude, or the time and altitude of its injection, requiring additional analysis to quantify the SO_2_ emission rates (Theys et al. 2013). Here we present a novel forward-trajectory approach using PlumeTraj (Pardini et al. 2017, 2018; Queißer et al. 2019; Burton et al. 2021; Esse et al. 2023a, b; Hayer et al. 2023) in which we initialise atmospheric trajectories from the source and track them as they disperse downwind. This is a significant update in methodology, and so the processing steps will be laid out below.

### PlumeTraj

#### Plume selection

The first step in reconstructing the SO_2_ emission is to identify which orbits to analyse and to filter the orbit data for plume pixels. All TROPOMI orbits that intersected with a given region of interest (18°N ≤ latitude ≤ 38°N, 35°W ≤ longitude ≤ 5°W) between 19 September–14 December 2021 were analysed, typically capturing 2–3 orbits each day. The best orbit, or combination of orbits, for each day was selected manually to ensure that the full plume was imaged while avoiding double counting emissions present in multiple orbits. The SO_2_ detection flag available within the TROPOMI data was used to define the plume pixels within the region of interest (using any pixel flagged for SO_2_ regardless of specific flags within the TROPOMI files for likely source type).

#### Trajectory analysis

We release trajectories from the vent location at a range of altitudes (0.4–10 km asl in 0.2 km steps) every 30 min throughout the entire eruption, calculating them up to an age of 30 h. The trajectories were generated using the Hybrid Single-Particle Lagrangian Integrated Trajectory (HYSPLIT) dispersal model (Stein et al. 2015) using the National Oceanic and Atmospheric Administration (NOAA) Global Forecast System (GFS) 0.25° global meteorological data from the National Centers for Environmental Prediction (NCEP, available from https://www.ready.noaa.gov/archives.php). Any near real-time analysis applied uses the forecast GFS data; however, this is updated with the reanalysis data once available.

HYSPLIT takes the start location, altitude and time for each trajectory and calculates the predicted path through the atmosphere for a tracer air parcel using information on the wind field and atmospheric conditions provided by the meteorological data without any chemical processes or gravitational settling. Using a global meteorological model as the input is advantageous in that it can be applied anywhere in the world; however, it may not resolve small-scale features, especially around terrain. Given the scale of the plumes emitted by Tajogaite, this did not pose significant issues far downwind; however, close to the source, this may lead to errors in the trajectory paths.

Once released, these trajectories will diverge downwind of the source due to wind shear in the atmosphere. We construct an array of regular rectangles in altitude–time space, defined by the four adjacent release times and altitudes of the trajectories above the vent, and map these into an array of irregular polygons in latitude–longitude space, with the vertices given by the positions of the four trajectories at the time of measurement by TROPOMI (see supplementary figure S1). Any polygon that intersects a given TROPOMI pixel is flagged as a potential solution for that pixel, with adjacent intersecting polygons grouped together. Often there will be multiple solution groups as releases at different times and altitudes may both intersect the pixel; in this case, additional information is used to select the preferred solution. For this eruption, we take the solution closest to a target injection altitude of 3 km asl, chosen as a representative altitude derived from webcam footage throughout the eruption (Bonadonna et al. 2022). Uncertainties in the injection time, injection altitude and plume altitude are calculated by propagating the uncertainties from the grid spacing in trajectory release times and altitudes with the standard deviation of solution time and altitudes for the selected solution group.

This implementation of PlumeTraj is advantageous over previous methods which used independent trajectories and no temporal constraint, as it is more robust to spurious solutions caused by discontinuities in the wind field. This is because it incorporates both time and altitude information of the four trajectories used to define the polygons, whereas previously PlumeTraj only used the altitude information. The same method could be applied with back-trajectories, by defining a series of polygons along the back-trajectories for each pixel and finding those that intersect the vent location and mapping these onto the altitude–time emission grid. However, a forward-trajectory approach is now preferred as this drastically reduces the number of trajectories calculated (48 release times per day in forwards mode versus up to tens of thousands in backwards mode, depending on the plume size).

### SO_2_ emission reconstruction

The sensitivity of TROPOMI to SO_2_ is dependent on the altitude at which the gas is located. This is not known at the time of measurement, so three vertical column densities (VCDs) assuming 1-km-thick box profiles centred at 0.5 km above ground level, 7 km above sea level and 15 km above sea level are provided (in addition to a fourth product calculated assuming a polluted scene, which is not used for volcanic applications). Using the plume altitude calculated by PlumeTraj, we calculate a corrected VCD by linearly interpolating between the three box-profile Air Mass Factors (which are used to convert the measured slant column density to a VCD), to the plume altitude. The error in the corrected VCD is propagated by combining the uncertainties in the box VCD values given by TROPOMI with the altitude uncertainty from PlumeTraj.

Once the corrected VCD is calculated, the SO_2_ emission time series can be reconstructed by summing the SO_2_ mass (found by multiplying the interpolated VCD by the pixel area) of all pixels that share the same injection time and altitude. Note that if a pixel intersects adjacent solutions, then the mass is divided equally between these. The uncertainty in the SO_2_ emission is calculated by summing the uncertainties of the VCDs of contributing pixels for each emission grid point. Only pixels that are older than 3 h but younger than 27 h are used in the emission calculation. This avoids double counting emissions on multiple days while avoiding the influence of a systematic decrease in emission rate close to the overpass time due to high concentrations of SO_2_, ash and liquid aerosols in the near-field plume, as well as issues close to the source due to the spatial resolution of the GFS meteorological data used.

This provides an SO_2_ emission intensity (the strength of the SO_2_ emission as a function of time and altitude) throughout the whole eruption. The total emission rate is calculated by integrating the emission intensity across all altitudes. A daily emission rate is calculated as the mean of all emission rates on a given day above 0.4 kt day^−1^ (or 5 kg∙s^−1^), chosen to avoid averaging over periods with no detectable emissions towards the end of the eruption when less than 24 h of emission are visible in the TROPOMI imagery. The uncertainty on the daily emission is calculated from the upper and lower uncertainties of the emission rates for that day.

### Cumulative emissions

Calculating the cumulative emission of SO_2_ throughout an eruption can provide useful insights into the evolution of eruptive activity. Using the time evolution of SO_2_ emissions as an eruption forecasting tool was trialled by fitting an exponentially decaying emission rate to the cumulative emissions. The cumulative emitted mass was modelled as:

1 $${M}_{{SO}_{2}}\left(n\right)=\sum_{i=1}^{n}{\phi }_{0}\cdot {e}^{-{~}^{i}\!\left/ \!{~}_{\tau }\right.}$$

where ${M}_{{SO}_{2}}(n)$ is the total emitted SO_2_ up to day $n$ of the eruption in kt, ${\phi }_{0}$ is the initial daily emission rate in kt·day^−1^, $i$ is the eruption day and $\tau$ is the decay time constant in days. Note that both ${\phi }_{0}$ and $\tau$ were left as free parameters in the fit. The uncertainty on the cumulative emissions is calculated as the sum in quadrature of the individual daily SO_2_ emission uncertainties. It is important to note that most physical processes impacting the satellite retrieval of the SO_2_ column densities (cloud cover, plume dispersal, chemical processing of SO_2_ to sulphate, presence of volcanic ash) will lead to underestimation of the emission rate. For this reason, we also calculate the cumulative emitted SO_2_ for the upper uncertainty limit on each day, which should encompass any underestimation caused by these factors.

The error on the fit was calculated by propagating the reported uncertainties of the fitted parameters as:

2 $$\Delta {M}_{S{O}_{2}}\left(n\right)= \sum_{i=1}^{n}\sqrt{{\left({\Delta \phi }_{0}\cdot {e}^{{~}^{-i}\!\left/ \!{~}_{\tau }\right.}\right)}^{2}+{\left(\frac{\Delta \tau \cdot i\cdot {\phi }_{0}\cdot {e}^{{~}^{-i}\!\left/ \!{~}_{\tau }\right.}}{{\tau }^{2}}\right)}^{2}}$$

where $\Delta$ signifies the absolute uncertainty on a value.

### Limitations

PlumeTraj has some limitations and assumptions that can impact the results:

1. **Accuracy of satellite retrievals:** TROPOMI’s sensitivity to SO_2_ lowers with decreasing plume altitude. Other factors, such as the presence of significant aerosol in the plume or cloud cover, may also obscure emissions. This can hamper the retrieval, leading to (usually) underestimations of the SO_2_ mass and some of the plume dropping below the detection threshold as it disperses downwind. These factors will be particularly important for the later stages of the eruption where the SO_2_ emission is weaker. To combat this, the daily emission rates are calculated for all times above 0.4 kt day^−1^ and we also calculate an upper cumulative mass limit based on the upper uncertainty limit each day.
2. **Corrected VCD calculation:** to calculate the height-corrected VCD, we interpolate between the three 1-km-thick box AMFs. It would be preferable to recalculate the updated AMF directly; however, the information required to do this is not provided in the TROPOMI files due to data storage constraints. To do this in the future, we could incorporate the lookup table used in the operational processor to fully recalculate the weighting function.
3. **Use of global meteorological data:** the meteorological data used has a coarse spatial resolution (~ 25 km at the equator) and will not resolve the detail of terrain over La Palma. To improve this in the future, we could use local mesoscale meteorological models to drive the trajectories. This is the subject of ongoing work. Additionally, using an ensemble of meteorological models to better constrain the accuracy of the model wind fields would improve quantification of the model uncertainties.

### Conduit model

To determine which processes might best explain the observed exponential decay in SO_2_ emission rate, we applied a 1D steady-state magma ascent model produced by La Spina et al. (2022). This model includes the main processes occurring during magma ascent, such as crystallisation (including disequilibrium crystal growth), exsolution, outgassing, rheological evolution, thermodynamics (and therefore temperature evolution) and fragmentation. The governing and constitutive equations of the magma ascent model are those reported in La Spina et al. (2022), but with a crystallisation model designed for the Tajogaite eruption, derived using the average melt inclusion data from Burton et al. (2023). The melt compositions used for the fitting of the crystallisation model are reported in Supplementary Table S1. Among all melt inclusions available, the average post-entrapment corrected melt inclusion data has sulphur (S) at 3290 ppm and potassium (K) between 1.2 and 1.5 wt%, as these should be representative of the most primitive and least degassed magma.

Following La Spina et al. (2022), the equilibrium total crystal contents are expressed as a function of temperature, pressure and dissolved water content. With these assumptions, the equilibrium mass fraction of crystal phase ${x}_{c}^{l,eq}$ is computed using the polynomial function:

3 $${x}_{c}^{l,eq}\left({P}^{*},{T}^{*},{x}_{d}^{*}\right)={\xi }_{1}{\left({P}^{*}\right)}^{2}+{\xi }_{2}{\left({T}^{*}\right)}^{2}+{\xi }_{3}{\left({x}_{d}^{*}\right)}^{2}+{\xi }_{4}{P}^{*}{T}^{*}+{\xi }_{5}{T}^{*}{x}_{d}^{*}+{\xi }_{6}{P}^{*}{x}_{d}^{*}+{\xi }_{7}{P}^{*}+{\xi }_{8}{T}^{*}+{\xi }_{9}{x}_{d}^{*}+{\xi }_{10}$$

where ${P}^{*}$ is the magma pressure (expressed in bars), ${T}^{*}$ is the temperature (expressed in Celsius degrees) and ${x}_{d}^{*}$ is the dissolved water concentration (expressed in weight percent). By fitting the previous equation over a large range of data obtained at different pressures, temperatures and water contents with alphaMELTS 2.1 (https://magmasource.caltech.edu/alphamelts/version2.php, Gualda et al. 2012), it is possible to calculate the fitting parameters ${\xi }_{j}$.

The data for the fitting has been obtained by running alphaMELTS 2.1 in combination with DAKOTA (Design Analysis Kit for Optimisation and Terascale Applications) (Adams et al. 2021), an open-source software toolkit developed at Sandia National Laboratories. DAKOTA provides a flexible and extensible interface between analysis codes and iterative systems analysis methods, such as uncertainty quantification, sensitivity analysis, optimisation and parameter estimation. We performed more than 800,000 alphaMelts simulations assuming a pressure, temperature and water content within the following ranges: 1–3500 bar, 1000–1200 °C and 0.1–6 wt%, respectively. The oxygen fugacity used for the calculation is NNO. The fitting coefficients computed for the La Palma crystallisation model are reported in Supplementary Table S2.

The average melt inclusion composition has also been used for the rheological model of the crystal-free bubble-free melt, as indicated in La Spina et al. (2022) and Giordano et al. (2008). The effect of the presence of crystals on the rheology has been taken into account following Costa et al. (2009) and Vona et al. (2011), whereas the effect of the bubbles has been considered using the low capillary number formulation from Llewellin et al. (2002) and Mader et al. (2013). Finally, we adopted the solubility model and solubility parameters reported in Arzilli et al. (2019).

The model has, of course, some limitations, the main one being the assumption of a cylindrical conduit with constant radius. When dealing with a complex volcanic plumbing system this is certainly a limitation, but the likely evolution of the plumbing system towards a conduit-like connection between reservoir and eruption justifies an assumption of simple geometry. We propose therefore that, to first order, this model can provide insights into the main parameters that can control the observed mass eruption rate at the surface. Although the 1D steady-state model has no time variation, we can explore the possible evolution of the magmatic system by simulating multiple possible conditions that could explain the evolution of the SO_2_ emissions seen.

## Results

### *SO*_*2*_* emissions*

The first TROPOMI orbit to properly detect SO_2_ from Tajogaite occurred on 20 September. There are some pixels with visible SO_2_ enhancement the previous day; however, these were measured only 30 min after the eruption onset and did not pass the TROPOMI thresholding. This shows that there was no precursory degassing detected. Additionally, no SO_2_ was detectable by TROPOMI after the eruption end, though SO_2_ was detectable by ground-based measurements after the eruption (Global Volcanism Program 2021), with emission rates less than 1 kt·day^−1^ (metric tons, 1 t = 1000 kg).

**Fig. 2:**
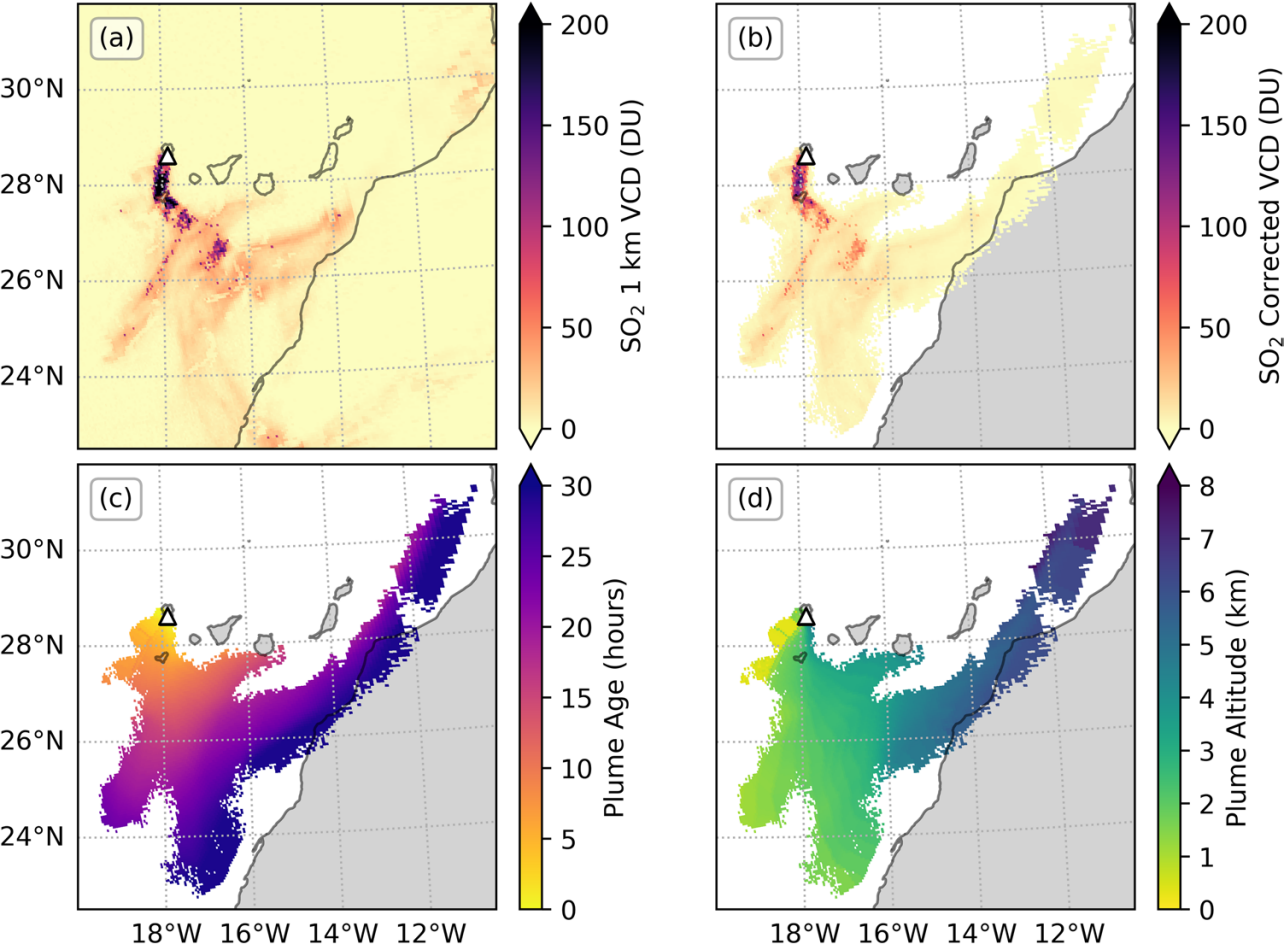
Example of PlumeTraj results. **a** The original 1 km VCD given in the operational TROPOMI product, **b** the altitude corrected VCD value, **c** the plume age at the time of measurement and **d** the plume altitude at the time of measurement. The location of the vent is given by the white triangle. The overpass time was approximately 14:35 (UTC) on 10 October 2021 (orbit number 20688)

The observed SO_2_ plumes extended for hundreds of kilometres from the volcano, dispersing in various directions on different days depending on the local wind field. Figure 2 gives an example of the TROPOMI SO_2_ imagery and PlumeTraj results for an overpass on 10 October, showing the VCD calculated assuming all gas was between the ground and 1 km, the VCDs corrected for the retrieved height of each pixel, plume age and plume altitude for selected plume pixels. VCD values are reported in Dobson Units (DU, 1 DU = 2.69 × 10^16^ molecules·cm^−2^). Note that the most distal regions of the plume do not have solutions as they are older than 30 h, and so beyond the range of the modelled trajectories. We highlight how the two “arms” of the plume have different altitudes, showing the impact of wind shear in the atmosphere taking these portions of the plume, injected at different heights, in different directions over time.

**Fig. 3:**
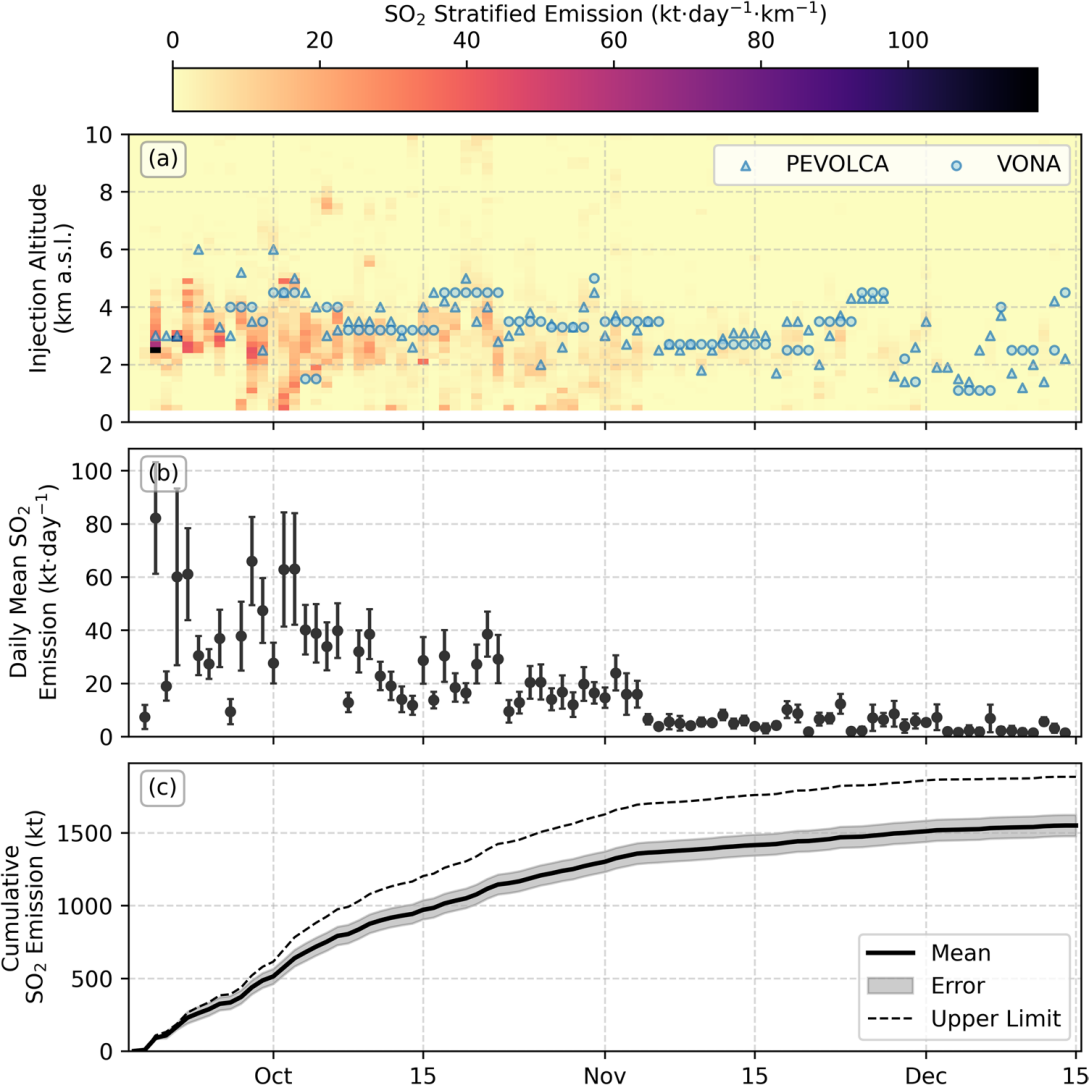
SO_2_ emissions from the Tajogaite eruption. **a** The total daily stratified emissions as a function of altitude (colormap) with plume altitudes from the PEVOLCA and VONA (blue triangles and circles, respectively) reports for comparison (data from Bonadonna et al. (2022)), **b** the daily mean SO_2_ emission rates calculated by PlumeTraj and **c** the cumulative emitted SO_2_ for the mean (solid line, uncertainty given by shaded region) and upper limit of daily measurements (dashed line)

The calculated emission results are shown in Fig. 3. The daily altitude-resolved emissions are given in Fig. 3a, with plume height measurements reported by the Plan de Emergencias Volcánicas de Canarias (PEVOLCA) and Volcano Observatory Notice for Aviation (VONA) reports, and compiled by Bonadonna et al. (2022). Also shown are the daily mean emission rates (Fig. 3b), and the cumulative emitted SO_2_ mass (Fig. 3c). The impact of the time window used was assessed by calculating the daily emissions for a range of windows (see supplementary figure S2), finding that all results agreed within uncertainty.

The injection altitudes calculated by PlumeTraj agree well with the plume-top heights from PEVOLCA and VONA, with the reported values closely tracking the upper limit of the SO_2_ injection altitudes (representing the top of the visible plume). The emissions on 20 September were high (~ 80 kt·day^−1^) but decreased significantly over the following days. Very low emissions were measured on 27 September, coincident with a 10-h pause in activity (Romero et al. 2022). The SO_2_ emissions reached a new peak value of ~ 70 kt·day^−1^ on 29 September, after which the measured emissions decreased with time until the end of the eruption. The total measured SO_2_ from the eruption calculated by PlumeTraj is 1.6 (± 0.1) Mt, with an upper limit of 1.9 Mt. This is in agreement with another TROPOMI-derived estimate from the MOUNTS system of 1.8 ± 0.9 Mt (http://www.mounts-project.com/; Valade et al. 2019; Milford et al. 2023) and somewhat lower than the value of 2.4 ± 0.4 Mt from analysis of melt inclusions (Dayton et al. 2024).

Some SO_2_ is emitted at altitudes lower than the vent, which can either be due to the coarse meteorological data not resolving the landmass of La Palma or be from emissions from the lava flow. Burton et al. (2023) demonstrate that the oxidised nature of the melt supplying the Tajogaite eruption favoured a high sulphur solubility, allowing significant degassing of sulphur from the lava flows after emission.

### Forecasting emission evolution

**Fig. 4:**
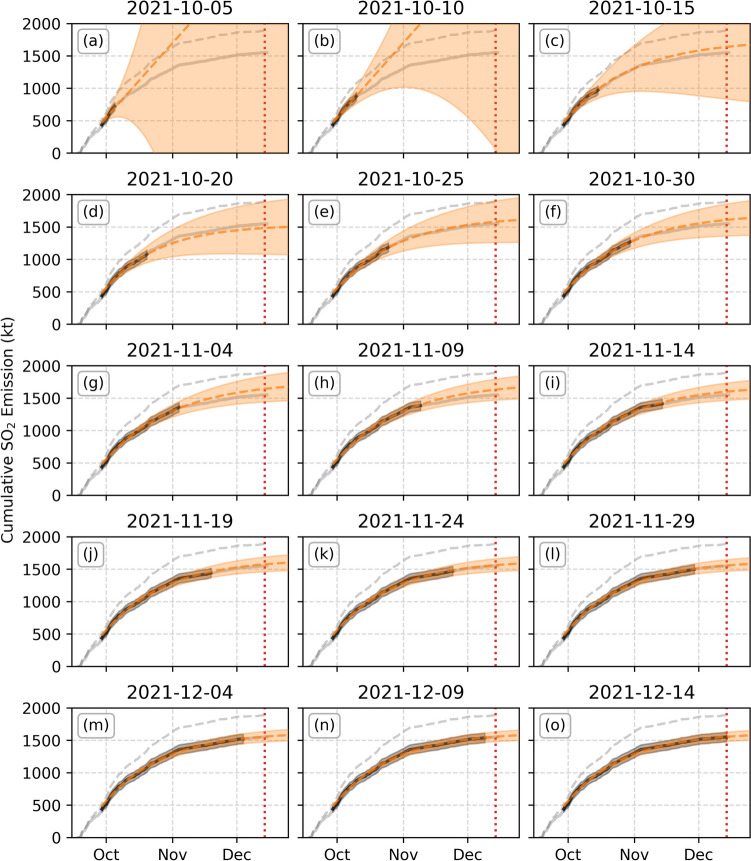
Cumulative and forecasted SO_2_ emissions at different stages throughout the eruption. Full cumulative emission data is given in the light grey solid line, upper limit cumulative emission is given by the grey dashed line, cumulative data used for each fit is given in black with shaded grey regions giving uncertainty. Fitted forecasts are given in the orange dashed line, with the fitted uncertainty in the pale orange shaded regions. The observed eruption end is marked by the red vertical dotted line

The cumulative emission trend (Fig. 3c) shows a roughly linear growth up to October, before an overall smoothly decreasing gradient until the end of the eruption. The emission rate was modelled as an exponential decay function from the day of maximum emission (29 September, excluding the initial high peak) with time constant $\tau$ (time in days to reduce to 36.8% of the current value) and initial SO_2_ emission rate ${\phi }_{0}$ (from the 29 September), fitted to the cumulative emission rate every 5 days and projected forward in time. The results are shown in Fig. 4.

**Fig. 5:**
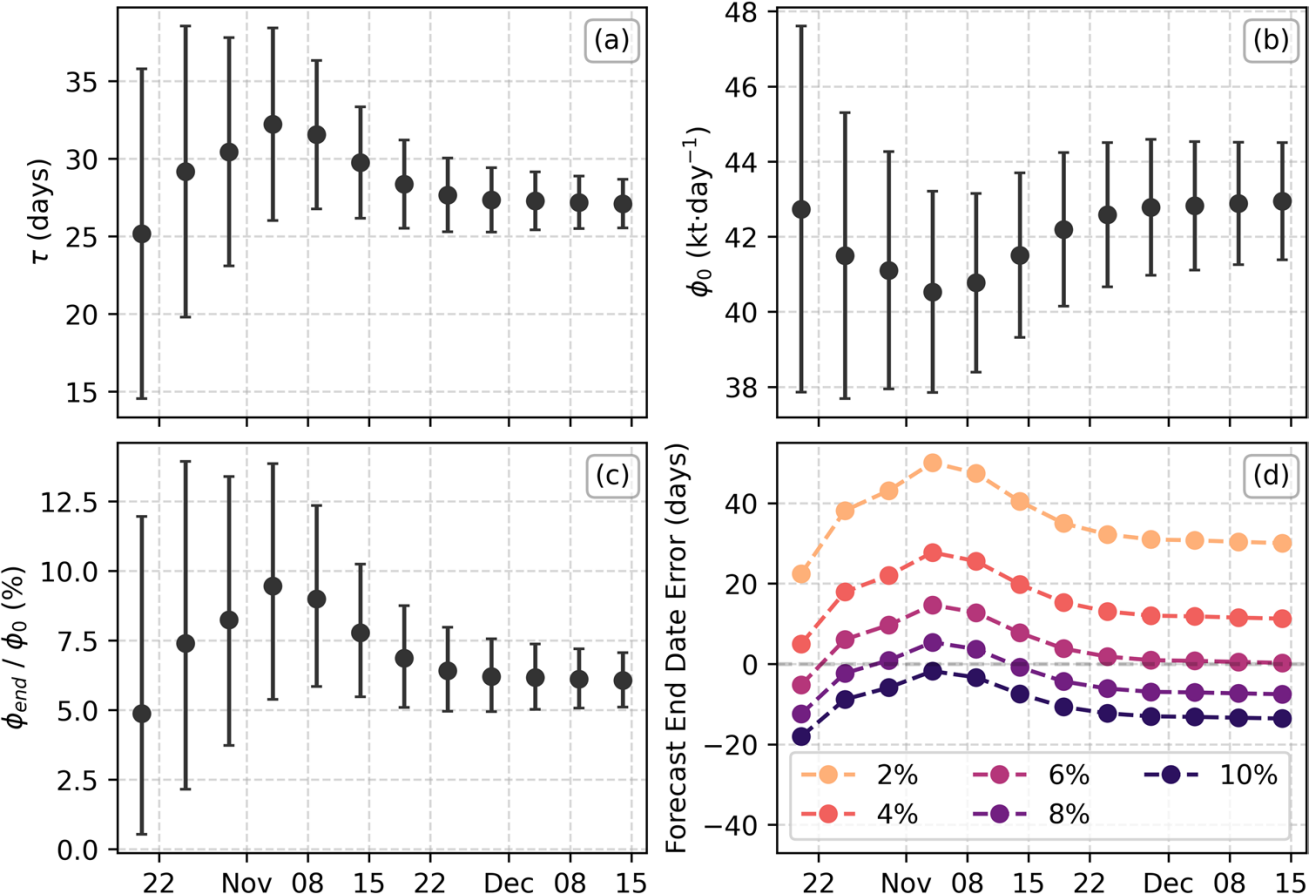
SO_2_ emission forecast results from 20 October. **a** Fitted decay constant, $\tau$; **b** fitted initial emission rate, ${\phi }_{0}$; **c** the final SO_2_ emission rate as a percentage of the initial emission rate, ${\phi }_{0}$; and **d** difference in forecasted and actual eruption end dates assuming different cut-off emission thresholds

The first three fits (up to 15 October) are not well constrained by the available data, providing fitted uncertainties of over 100%, but all subsequent fits produce consistent forecasts, with both $\tau$ and ${\phi }_{0}$ steady within fitted uncertainty (Fig. 5a, b). As expected, the fit uncertainty reduces with time as more data is included. It is also possible to calculate final emission rate as a percentage of the initial emission rate at the start of October (Fig. 5c). This shows a consistent value of roughly 6%, corresponding to a final emission rate of roughly 3 kt∙day^−1^. Finally, we investigate the ability to forecast the eruption end date using this data. The use of an exponential decay requires the definition of a cut-off threshold at which point the eruption can no longer sustain itself. Our best fit gives this value as 6%, so we varied this between 2 and 10% to investigate the sensitivity of the forecasted end date to this threshold (Fig. 5d).

These results show that the fitted SO_2_ emission evolution was consistent within uncertainty from 20 October onwards, with decreasing uncertainty as more data is included in the fit. Using a 6% limit, the forecasted eruption end is within 15 days of the true end for all times investigated, and all limits except 2% are within 30 days of the true end.

### Magma ascent modelling

**Fig. 6:**
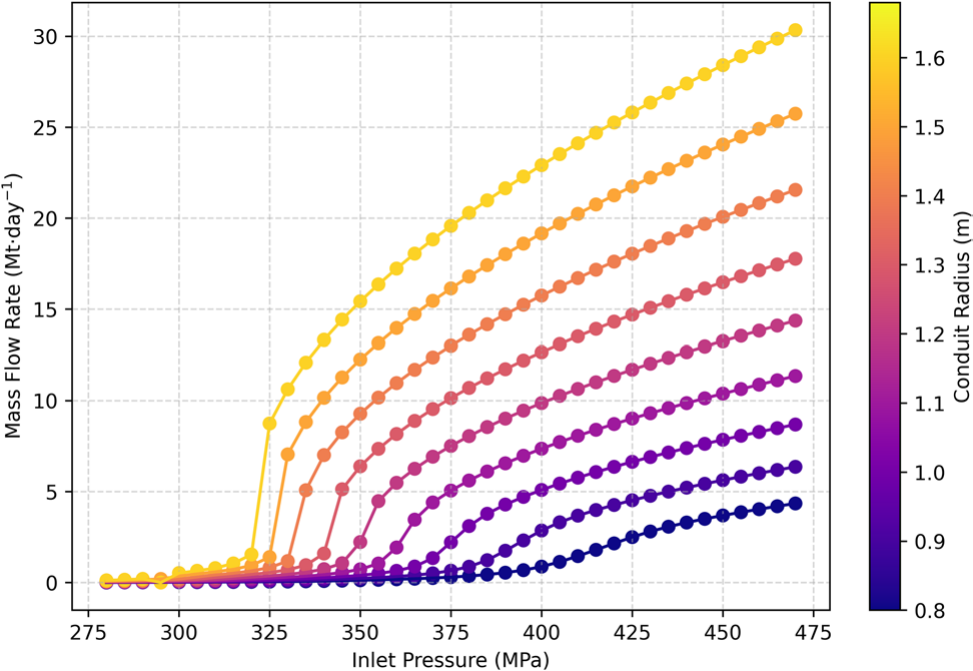
Results of magma ascent modelling. Each point represents a single model run, with the resulting magma mass flow rate on the *y*-axis, the inlet pressure on the* x*-axis and the conduit radius given by the colour

For the simulations of the Tajogaite eruption, we considered a 13-km-long cylindrical conduit, constrained by the depths of seismic energy release (D’Auria et al. 2022), with an inlet temperature of 1160°C, volatile contents of 3 wt% H_2_O and 4.5 wt% CO_2_ (Burton et al. 2023). Since at 13 km-depth the lithostatic pressure is ~ 350 MPa, to investigate the control of magma chamber pressure on the total mass flow rate, we considered a wide range of values across the lithostatic pressure, going from 280 to 470 MPa. The conduit radius was varied between 0.8 and 1.6 m to generate mass flow rates covering those observed during the Tajogaite eruption. The results of the modelling are shown in Fig. 6.

**Fig. 7:**
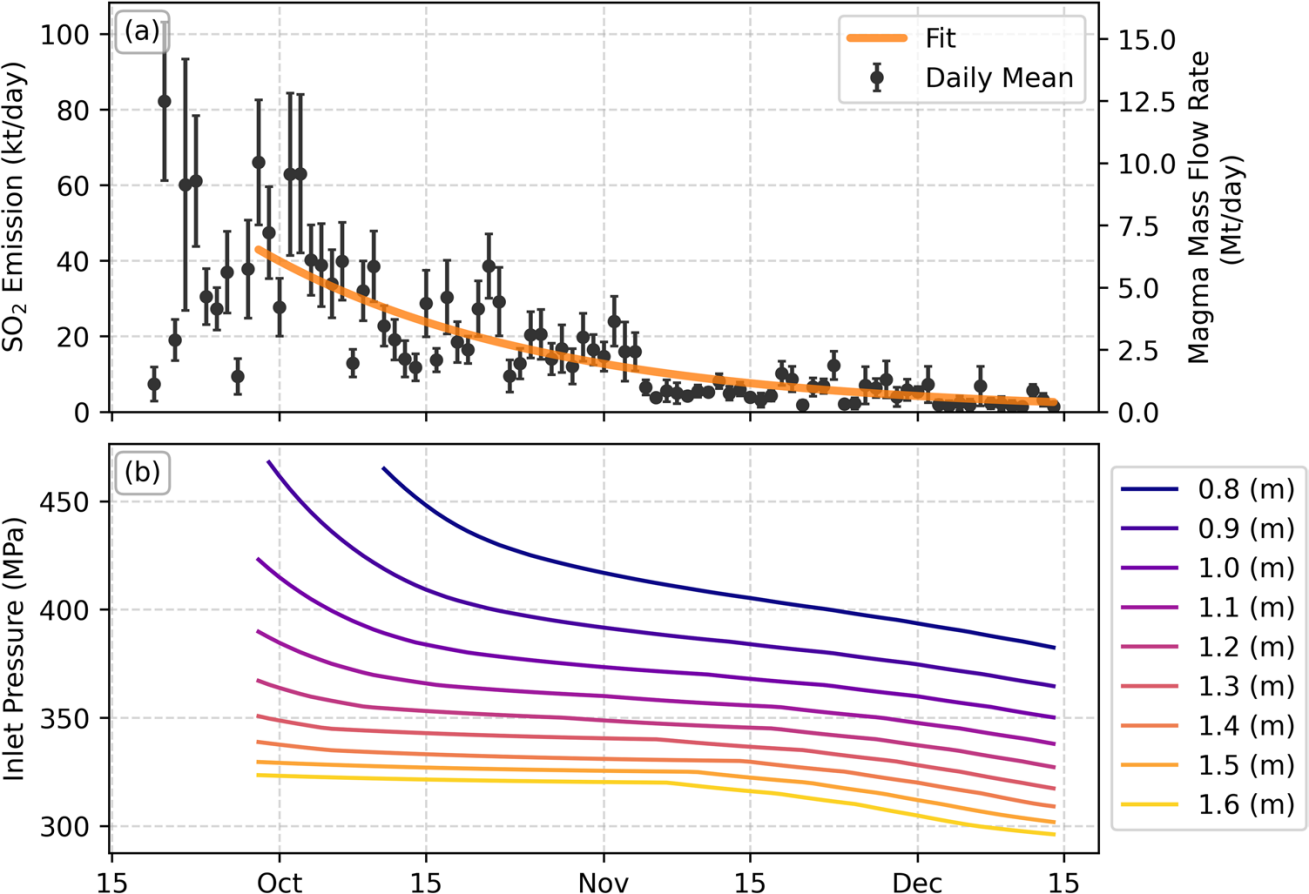
Calculated time evolution of the conduit condition from the magma ascent model. **a** The daily mean and final fitted SO_2_ emission rates with the corresponding magma volume flow rates. **b** The calculated conduit inlet pressures for given conduit radii

To explore the possible time evolution of the inlet pressure and conduit radius throughout the eruption, we converted the final fitted SO_2_ emission (Fig. 4o) to magma mass flow rates by scaling with a magma sulphur content of 3290 (± 390) ppm, measured from erupted melt inclusions (Burton et al. 2023). For each day in the eruption, we then determined the inlet pressure for given conduit radii by interpolating between the modelled mass flow rates to those calculated from the SO_2_ emissions. The results of these calculations are shown in Fig. 7.

## Discussion

In this paper, we have demonstrated the ability of a novel implementation of PlumeTraj to quantify the SO_2_ emissions throughout the entire Tajogaite eruption. This information was communicated in near real time to the monitoring agencies and used to inform the eruption response. The SO_2_ emissions from this eruption can be split into two phases, before and after 29 September: the first phase has dynamic and variable emissions with no clear trend with time, while the second has steadily decaying emissions. The initial very high emission (~ 80 kt∙day^−1^) on 20 September (Fig. 3b) may also suggest a pre-exsolved gas phase that developed before the eruption began. We propose that this is the surface expression of the evolution of the initial fissure system which was in place at the beginning of the eruption to a narrow conduit-like geometry, more directly linked to the deep system. The focussing of an eruptive fissure into localised and discrete vents is commonly seen for basaltic eruptions (Jones et al. 2017; Witt et al. 2018; Neal et al. 2019; Pedersen et al. 2022). The conduit geometry then directly coupled the deeper magma reservoir pressure with the magma eruption rate, producing an exponential decay in emissions with time, supported by the coincident reactivation of deeper (~ 20–25 km) seismicity (D’Auria et al. 2022). This suggests that the eruption was driven by the simple draining of the magma reservoir (Wadge 1981; Bonny and Wright 2017). This exponential pattern mirrors those observed in the lava effusion rate (Plank et al. 2023) and deflation measurement by GPS (Charco et al. 2024) for this eruption, and allowed a robust hindcast of the evolution of the eruption from 20 October onwards.

This opens the possibility of using SO_2_ emission rates to forecast the durations of future eruptions; however, this will require more studies of past events to determine what controls the end point of activity and how prevalent this pattern is across a wide range of eruption styles and tectonic settings. Previous work on the use of lava effusion rates to forecast eruption durations found that roughly 30% of observed eruptions followed an exponentially decaying model (Bonny and Wright 2017). A similar study for SO_2_ emissions would be a key benchmark for using this systematically in the future. It is worth noting that deviations from this model would lead to the generated forecast failing; however, this would still help identify when an eruption is not characterised by a simple draining of a magma chamber, reflecting more complex processes at work that could be further investigated. It is expected that this is more likely the case for volcanoes in subduction zones or open vent volcanoes, where a single magma body is less likely to form.

One weakness of using an exponential decay model is that there is no defined end point, requiring a threshold value to be set at which point the eruption can no longer sustain itself. We investigated the sensitivity of the eruption end date to different thresholds between 2 and 10% of the peak emission rate, finding a maximum of 50 days difference from the true end date at the end of the eruption, but with most estimates within 30 days (Fig. 5d). This highlights that the threshold value used to forecast the eruption end would be a key source of uncertainty in the case of future eruption forecasts.

Although the overall evolution of the SO_2_ emissions was well characterised by an exponential decay, some shorter-term variations in the SO_2_ emission behaviour are seen. Two peaks in SO_2_ emissions are seen around 20 October and 1 November (Fig. 3b), coinciding with peaks in measured plume altitude (Fig. 3a), suggesting short-term increase in the magma supply rate. This could reflect periods of recharge of the shallow magma chamber from the deeper source identified from seismic data (D’Auria et al. 2022; Ubide et al. 2023). We also see a sustained dip in SO_2_ emissions in early November, coinciding with a jump in deflation (Charco et al. 2024) and a shift in magma geochemistry (Ubide et al. 2023), indicating some key change in the magmatic system which would merit further investigation combining these datasets.

Using a 1-D numerical model of magma ascent, we were able to investigate the possible driving physical mechanisms of the decay in emissions seen (Fig. 7b). Both a decreasing inlet pressure and/or shrinking conduit radius could explain the drop in the observed SO_2_ emissions. At larger radii (> 1.3 m), a ~ 20–30-MPa pressure decrease is sufficient to reproduce the variation in mass flow rate, while if the pressure is fixed then a decrease in conduit radius of ~ 30–50 cm is sufficient. We suggest the most likely physical explanation supported by our results is a steady decrease in the inlet pressure as magma feeding the eruption is drained from the chamber, potentially combined with some thinning of the conduit radius due to deposition of magma during ascent. This is in good agreement with observations of deformation from GPS data (Charco et al. 2024) and aligns with previous models of a simply draining magma reservoir (Wadge 1981).

While emptying of a magma reservoir is consistent with SO_2_ emissions and ground deformation evolution during the eruption, it is not the only possibility to explain our observations. Significant changes in magma geochemistry were also observed throughout the eruption (Ubide et al. 2023), potentially influencing magma viscosity, flow dynamics and therefore SO_2_ emission rates. The links between gas flux, gas composition, eruptive behaviour and geochemical evolution require further investigation and are part of planned future work.

These results have significant implications for future volcanic eruptions, demonstrating the power of satellite-derived SO_2_ emissions for informing eruption response and understanding the driving magmatic processes. All required data are available freely globally in near real-time, providing a powerful tool for volcano monitoring in the future.

## Supplementary Information

Below is the link to the electronic supplementary material.

Supplementary file1 (XLSX 11 KB)

Supplementary file2 (PDF 545 KB)

## Data Availability

The TROPOMI SO_2_ data used in this study can be accessed from the Copernicus Data Space Ecosystem (https://dataspace.copernicus.eu/). The GFS meteorological data is available from NOAA (ftp://arlftp.arlhq.noaa.gov/pub/archives/). All PlumeTraj products, ascent modelling results and supplementary tables presented in this paper are available online (10.48420/26161543).
