# Supplementary material for: Forecasting the evolution of the 2021 Tajogaite eruption, La Palma, with TROPOMI/PlumeTraj-derived SO2 emission rates
Source: Bull Volcanol. 2025 Feb 26;87(3):20. doi: 10.1007/s00445-025-01803-6 (PMC11865176; doi:10.1007/s00445-025-01803-6)
Supplement: Supplementary file 2 — Supplementary file2 (PDF 545 KB) [file 445_2025_1803_MOESM2_ESM.pdf]

Forecasting the evolution of the 2021 Tajogaite eruption, La Palma, with satellite derived SO_2 emission rates – Supplementary Information

B. Esse[1], M. Burton[1,2], C. Hayer[3], G. La Spina[2], A. Pardo Cofrades[1], M. Asensio-Ramos[4], J. Barrancos[4,5], N. Pérez[4,6]

[1] Centre for the Observation and Modelling of Earthquakes, Volcanoes and Tectonics, Department of Earth and Environmental Sciences, The University of Manchester, Manchester, UK

[2] Istituto Nazionale di Geofisica e Vulcanologia, Sezione di Catania, Italy

[3] HAMTEC for EUMETSAT, Darmstadt, Germany

[4] Instituto Volcanológico de Canarias (INVOLCAN), 38320 San Cristóbal de La Laguna, Tenerife, Canary Islands, Spain

[5] Grupo de Observación de la Tierra y la Atmósfera (GOTA). Universidad de La Laguna. Avda. Astrofísico Francisco Sánchez s/n, 38200, La Laguna, Tenerife, Spain

[6] Instituto Tecnológico y de Energías Renovables (ITER), 38600 Granadilla de Abona, Tenerife, Canary Islands, Spain

Corresponding author: B. Esse, benjamin.esse@manchester.ac.uk

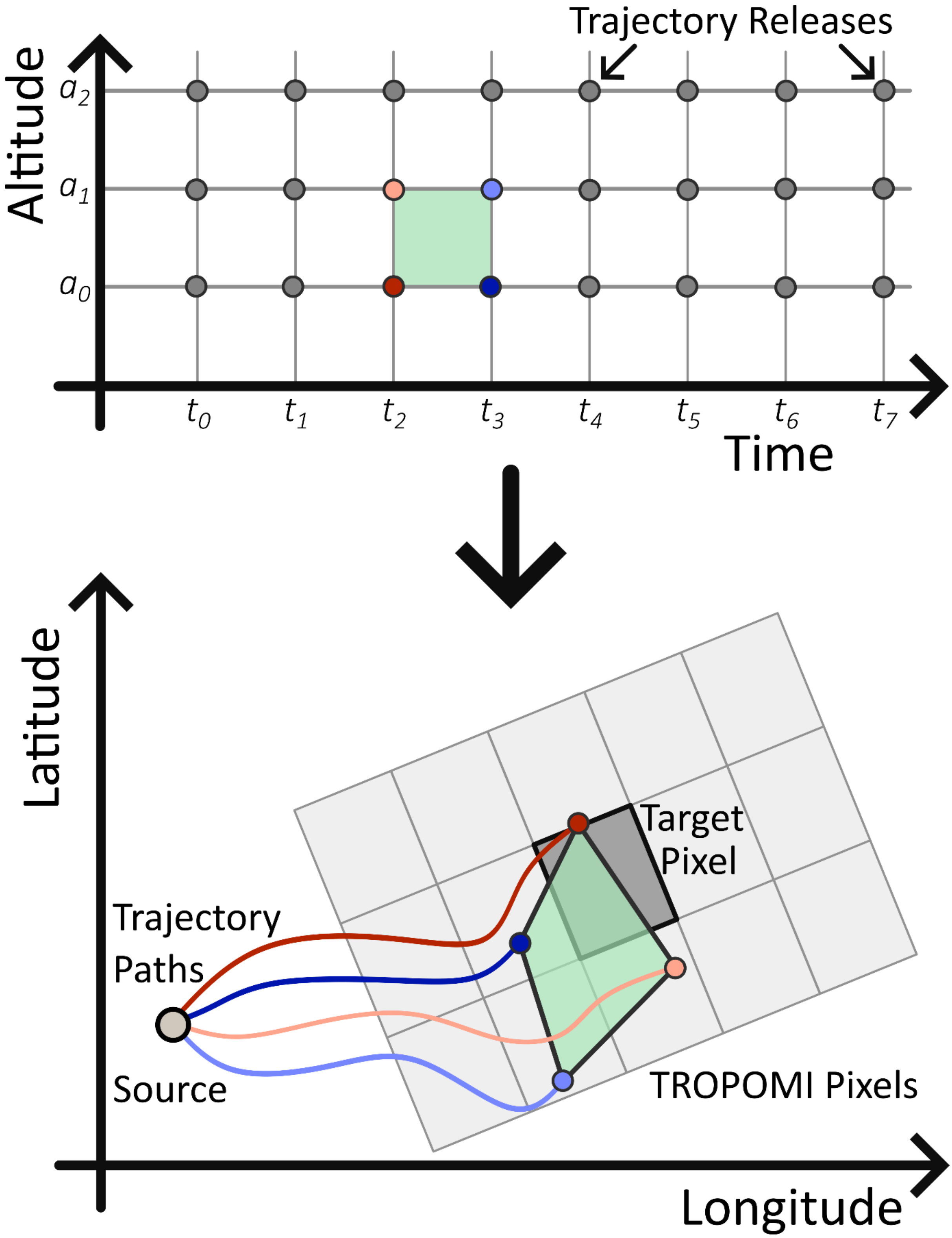

Fig. S1 Schematic of the updated PlumeTraj method. The locations of four adjacently released trajectories at the time of overpass of TROPOMI form the vertices of a polygon in latitude – longitude space. Any polygon intersecting a pixel are marked as a solution for that pixel.

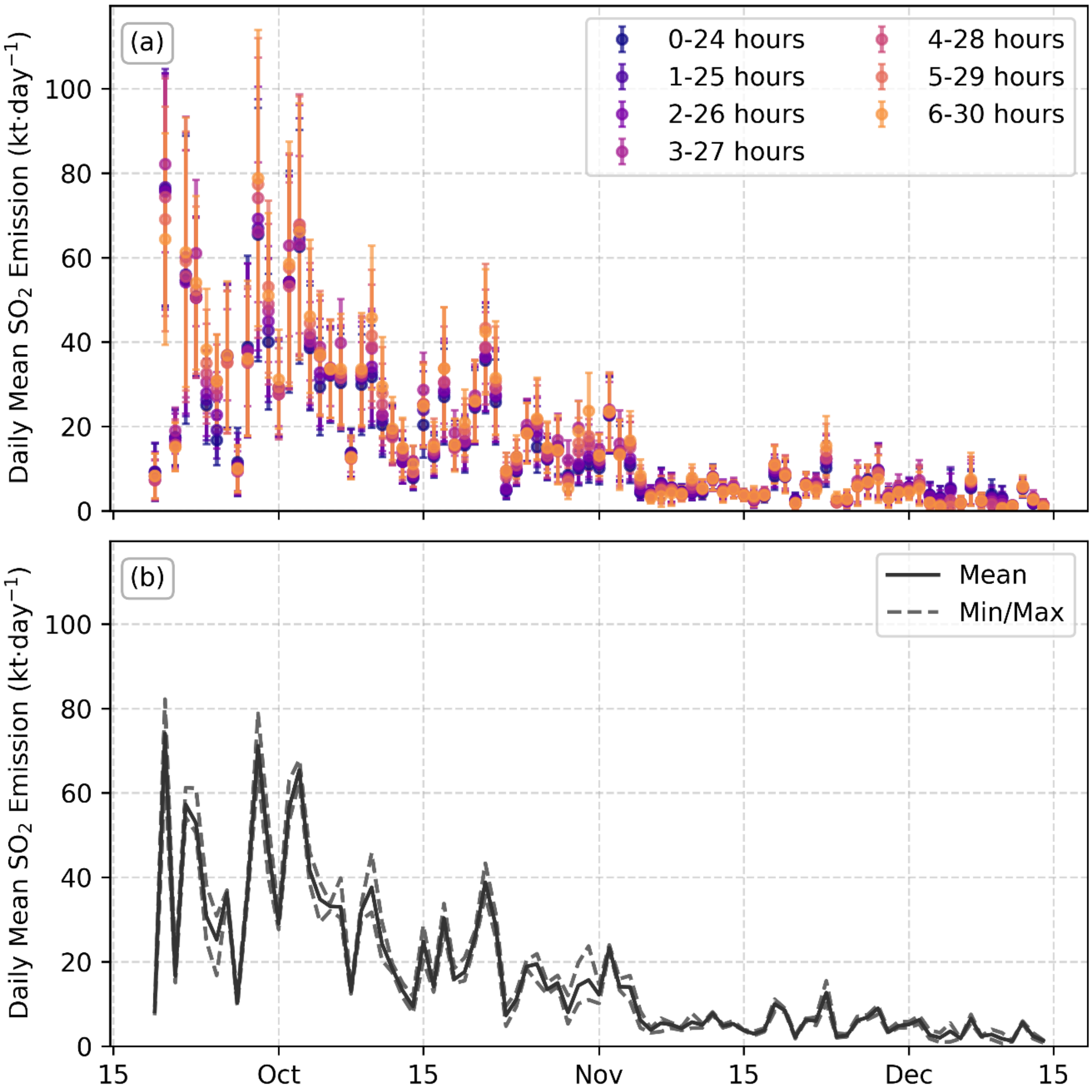

Fig. S2 (a) Mean daily SO_2 emissions calculated using differing time windows (starting from 0 – 6 hours old, each 24 hours wide). (b) mean of all values with minimum and maximum limits across the analysis.
